# Ultra-intense laser interaction with nanostructured near-critical plasmas

**DOI:** 10.1038/s41598-018-22147-6

**Published:** 2018-03-01

**Authors:** Luca Fedeli, Arianna Formenti, Lorenzo Cialfi, Andrea Pazzaglia, Matteo Passoni

**Affiliations:** Department of Energy, Politecnico di Milano, Via Ponzio 34/3, Milano, 20133 Italy

## Abstract

Near-critical plasmas irradiated at ultra-high laser intensities (*I* > 10^18^W/cm^2^) allow to improve the performances of laser-driven particle and radiation sources and to explore scenarios of great astrophysical interest. Near-critical plasmas with controlled properties can be obtained with nanostructured low-density materials. By means of 3D Particle-In-Cell simulations, we investigate how realistic nanostructures influence the interaction of an ultra-intense laser with a plasma having a near-critical average electron density. We find that the presence of a nanostructure strongly reduces the effect of pulse polarization and enhances the energy absorbed by the ion population, while generally leading to a significant decrease of the electron temperature with respect to a homogeneous near-critical plasma. We also observe an effect of the nanostructure morphology. These results are relevant both for a fundamental understanding and for the foreseen applications of laser-plasma interaction in the near-critical regime.

## Introduction

Laser interaction with near-critical plasmas (NCPs)^1,2^ at relativistic intensities, i.e. high enough (*I* > 10^18^ W/cm^2^) to accelerate electrons to relativistic energies in a single laser cycle, is studied for a wide range of applications, including advanced laser-driven sources (ions^3–15^, electrons^16–19^ and high-energy photons^20–23^), generation of dense pair plasmas^24^, generation of ion Weibel-mediated collisionless shocks^25^, High-order Harmonic emission^26^, plasma optics^11,27^ and inertial confinement fusion^28^. This interaction regime is characterized by a variety of physical processes, such as efficient laser absorption, excitation of bulk plasmons and relativistic solitons, strong self-focusing, channel formation, and betatron electron acceleration, which have been the object of extensive theoretical investigations^1,29–32^.

NCPs are defined as having an electron density close to the critical density *n*_*c*_(*λ*), which marks the transparency threshold for the propagation of an electromagnetic wave with wavelength *λ*. The critical density *n*_*c*_ is the electron density *n*_*e*_ which satisfies *ω*_*L*_ = *ω*_*p*_(*n*_*e*_), where *ω*_*L*_ = 2*π*/*λ* is the laser frequency and ${\omega }_{p}({n}_{e})=\sqrt{4\pi {n}_{e}{e}^{2}/{m}_{e}}$ is the plasma frequency (*m*_*e*_ is the electron mass, *e* is the elementary charge). Relativistic transparency^33^ allows a laser to propagate in a plasma beyond the classical transparency threshold marked by *n*_*c*_, as long as ${n}_{e} < {n}_{c}\sqrt{1+C{a}_{0}^{2}}$ (*C* = 0.5, 1, respectively for linear and circular polarization, ${a}_{0}=e{A}_{0}/{m}_{e}{c}^{2}$ is the normalized laser amplitude and *A*_0_ is the absolute value of the peak vector potential of the pulse). For the typical wavelengths of existing ultra-intense laser facilities^34^ (*λ* ~ 0.8 – 1 *μ*m), NCPs still represent a considerable challenge from the targetry point of view^35^: even a moderately ionized solid has an electron density two orders of magnitude greater than *n*_*c*_, while typical gas-jets provide electron densities well below *n*_*c*_. Near-critical electron densities can be obtained irradiating a dense cryogenic gas-jet^36^ or a pre-exploded target^4^. However, these techniques require complicated setups and lead to NCPs with non-finely controllable density gradients and compositions. This might be detrimental for schemes requiring a careful control of the electron density profile, such as laser-driven ion acceleration^37^ with targets consisting in a near-critical layer attached to a solid foil^8,10–13^ or *γ*-ray sources^23^ based on low-density channels embedded in a denser medium.

Low density nanostructured materials, such as nanotubes^38^, nanowires^39^ or foams^40–42^ allow to obtain NCPs without further complications for the experimental setup, potentially providing at the same time an enhanced control over the density profile of the plasma. These materials, while being near-critical on average, consist in alternating voids and solid-density structures. The typical scale-length of these density fluctuations might well be comparable with the laser wavelength (i.e. of the order of 1 *μ*m)^41^. Modern-day laser facilities can provide laser pulses with temporal contrast well below 10^−10^ at the few ps timescale and of $\sim {10}^{-6}$ at the 1 ps timescale^43–45^. Assuming a pre-heating of 100s eV, the characteristic expansion velocity of the plasma is $\sim 100$ nm/ps, hence in ∼1 ps the plasma has not enough time to fill the micrometric voids typical of low density nanostructures. Thus, a nanostructure might survive long enough to influence the interaction with the laser, especially at lower laser intensities, since the pre-pulses are less intense. This is confirmed by several experimental works carried out irradiating nanostructured targets at relativistic intensities^3,44,46,47^.

Despite the interest in NCPs and the growing literature regarding experiments carried out using nanostructured low-density materials^5,10–13,46–48^, the role played by the nanostructure is still poorly understood. Particle-In-Cell simulations^49^ provide a powerful tool to investigate laser-matter interaction at relativistic intensities, even with nanostructured targets. Nonetheless, laser interaction with nanostructured foams has been systematically investigated, very recently, only with 2D simulations^50,51^, which do not reliably allow to simulate realistic nanostructures and to study the effect of pulse polarization. In a 3D geometry realistic foams have been exclusively investigated in a specific configuration, in a work^12,13^ focused on ion acceleration. Few more papers include numerical simulations of low-density wire targets^46–48,52–55^, though not in a near-critical regime and often in a 2D geometry.

In this work, by means of an extensive 2D and 3D Particle-In-Cell simulation campaign, we investigate how realistic nanostructures can influence laser-plasma interaction in the near-critical regime. We consider ultra-short laser pulses with intensities in the range *a*_0_ = 5–45, which span the capabilities of current ultra-intense lasers, from table-top sub-100 TeraWatt systems up to state-of-the-art PetaWatt facilities. The specific case of *a*_0_ = 5 is particularly interesting since it corresponds to a relatively moderate intensity $I\sim 5\cdot {10}^{19}\,{\rm{W}}/{{\rm{cm}}}^{2}$, achievable in many facilities worldwide. Laser interaction with NCPs in such intensity regime could be appealing due to the increased absorption efficiency provided by the NCP, which could allow to observe or to enhance physical phenomena that would otherwise require higher laser intensities and, thus, larger and more expensive facilities. For this reason, for the lowest laser intensity case, we also investigate the effect of different nanostructure morphologies, considering two structures of wide experimental interest in the laser-plasma community and in materials science: foams^5,10,12,13^ and nanowires^46–48,52,53,55,56^. The relevance of the investigation of how these kinds of nanostructured materials behave in extreme electromagnetic fields goes beyond the generation of NCPs, being of interest for both fundamental science^57^ and specific applications^47,48^.

All the simulations have been carried out with an average density of 3 *n*_*c*_, which is both experimentally feasible with low density materials^41^ and well within the near-critical regime for the relativistic intensities considered here. Similarly to what was done by Robinson *et al*.^30^, in this work we define a NCP as a plasma whose electron density *n*_*e*_ satisfies $0.1\,{n}_{c} < {n}_{e} < {n}_{c}\sqrt{1+C{a}_{0}^{2}}$. If ${a}_{0}\gg 1$, as in our work, the upper limit for relativistic transparency can be reformulated as *S* < 1, where *S* = *n*_*e*_/*a*_0_*n*_*c*_ is the relativistic similarity parameter^58^.

## Results

**Figure 1:**
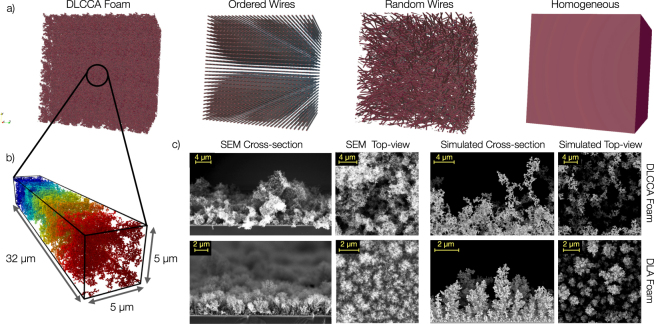
(**a**) Different plasma targets used in the simulation campaign: a foam obtained with a diffusion-limited cluster-cluster aggregation model, an ordered array of wires, a random wire assembly and a uniform slab. (**b**) Zoom of the foam structure. (**c**) Scanning Electron Microscopy (SEM) images of two foams with different morphology (upper vs. lower panels). For both foams, SEM cross-sections and top-views are compared with the simulated ones. The foam showed in the upper panel is well represented by a Diffusion-Limited Cluster-Cluster Aggregation model (DLCCA), whereas the foam showed in the lower panels is better represented by a simple Diffusion-Limited Aggregation model (DLA).

We simulate the interaction of an ultra-short ($\sim 30$ fs), relativistic (*a*_0_ = 5–45), Ti:Sapphire (*λ* = 0.8 *μ*m) laser pulse with the targets shown in Fig. 1. We consider foam targets made of nanospheres (radius of 40 nm) arranged in space according to two different models based on Diffusion Limited Aggregation^59^ (see Methods section for details). Qualitatively these models provide fractal-like structures: an aggregate of random clusters of nanoparticles (DLCCA foam) and a tree-like morphology (DLA foam). Both these structures are representative of realistic foam-like plasmas (see Fig. 1(c)). As far as nanowires (radius of 96 nm) targets are of concern, both an ordered array and a random assembly of wires are simulated. In all these cases the average electron density is 3*n*_*c*_ and the average filling factor is ~5%. Thus we explore scenarios ranging from near the transparency threshold (*S* = 0.6 for linear polarization) up to a moderately transparent regime (*S* = 0.067 for linear polarization).

We investigate the effect of the nanostructure morphologies on several quantities of experimental relevance, such as absorption efficiency, energy spectra and angular distribution of the emitted energetic particles.

We first discuss the effect of a foam-like nanostructure on the laser-matter coupling, comparing, at different laser intensities, some relevant quantities with those of a benchmark homogeneous plasma. In the second part we investigate different plasma nanostructures at fixed irradiation conditions. Lastly, we discuss the possible effects of the simulations dimensionality on the laser plasma-interaction with both homogeneous and nanostructured targets, which is of particular relevance for the numerical research in this field.

### Effect of nanostructure on laser interaction with NCPs as a function of *a*_0_

**Figure 2:**
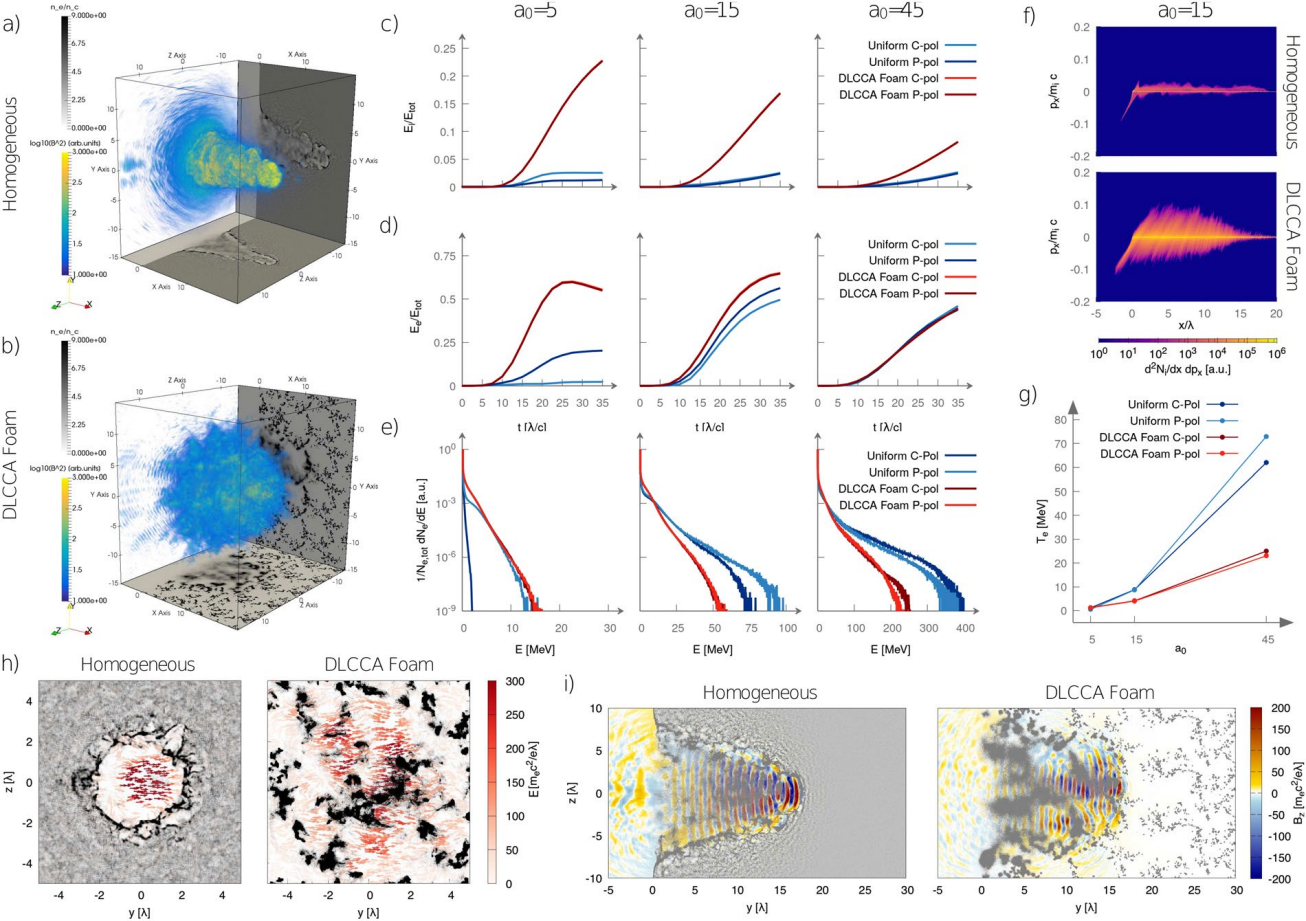
(**a**,**b**) Propagation of a P-polarized, *a*_0_ = 15, laser pulse in a uniform plasma and in a DLCCA foam plasma, respectively (snapshots at *t* = 30*λ*/*c*). Slices *y* = 0 and *z* = 0 of the electron density are represented in grey scale, while the square modulus of the magnetic field |B|^2^ is showed in color. (**c**,**d**) Time evolution of the absorption efficiency of laser energy into, respectively, ion and electron kinetic energy for uniform and foam targets, linear and circular polarization. (**e**) Electron energy spectra at time 30*λ*/*c* for uniform and foam targets, linear and circular polarization. (**f**) Projection of the ion phase space on the (*x*, *p*_*x*_) plane for uniform plasma (upper panel) and foam plasma (lower panel) (*a*_0_ = 15, P-polarization at time 40*λ*/*c*). (**g**) Temperature of the tail of the electron energy spectrum as a function of *a*_0_ for uniform and foam targets, linear and circular polarization. (**h**) Electric fields inside the plasma channel for *a*_0_ = 45 for a homogeneous plasma and a DLCCA foam respectively. The arrows show the direction of the electric field, while the color represents $\sqrt{{E}_{y}^{2}+{E}_{z}^{2}}$. The electron density is shown in greyscale. Results are shown at *t* = 30*λ*/*c* for the *x* = 15*λ* plane. (**i**) Cross-section at *z* = 0*λ* of the $\hat{z}$ component of the magnetic field for *a*_0_ = 45 and, respectively, a homogeneous plasma and a DLCCA foam plasma. The electron density is shown in greyscale, while the magnetic field is represented in color.

In this section we investigate the main differences in laser-plasma coupling with either homogeneous and nanostructured plasmas. A first qualitative view of the interaction is given in Fig. 2(a and b), which show that the pulse propagation occurs differently in homogeneous and nanostructured plasmas.

The nanostructured plasma allows for a stronger laser energy absorption and a much higher conversion efficiency of laser energy into ion kinetic energy. As shown in Fig. 2(c), the ion energy absorption remains well below 5% in all the homogeneous plasma cases, while it is remarkably enhanced with foams, up to 20% for *a*_0_ = 5. This is a direct consequence of the explosion of the nanospheres constituting the nanostructured plasmas, whose signature can be found in the ion phase space, as shown in Fig. 2(f). Both these effects are more relevant at the lowest intensity. The enhancement of laser absorption at *a*_0_ = 5 can be explained considering that the plasma is close to the transparency threshold. In the case of a uniform plasma, a denser layer is rapidly generated at the pulse front, hindering further propagation and increasing reflection. Conversely, a nanostructure lets the laser propagate through its voids deeper in the target, enhancing absorption. The higher absorption efficiency of laser energy into ion kinetic energy can be explained considering that laser energy is directly transferred to the electrons of the plasma. Indeed, due to their higher inertia, ions acquire energy only through the electric fields generated by charge separation. The local plasma density for the DLCCA foam target is ∼20 times higher than for the uniform plasma, so that significantly more intense electric fields are generated when electrons are blown away by the laser.

Figure 2(d) shows that, at the lowest intensity, the nanostructured plasma leads to a doubled electron energy absorption if compared to the homogeneous one. This absorption enhancement decreases at *a*_0_ = 15 and becomes negligible at *a*_0_ = 45 (the curves of energy absorption over time are almost superimposed in this case). This is due to a faster homogenization of the nanostructures at higher laser intensities. Simulations show that at the lowest intensity, the nanostructures are still clearly distinguishable after 20 laser cycles, while at the highest intensity only a weak signature of the original structure is visible. In order to have a better insight of the electron heating we also analyzed the electron energy spectra (shown in Fig. 2(e)). As a general feature, the foam plasma allows for a more efficient acceleration of mildly energetic electrons (i.e. with a kinetic energy $\lesssim 0.1{E}_{\mathrm{cut}-\mathrm{off}}$, where E_cut-off_ is the cut-off energy). On the other hand, the plasma nanostructure has a detrimental effect on the acceleration of the high-energy electrons. At *a*_0_ = 5 the effect is rather weak and the spectra are comparable, while at *a*_0_ = 15 and *a*_0_ = 45 they exhibit great differences, being the electron cut-off energies significantly reduced with foams. This also affects the mean kinetic energy of the most energetic component of the electron spectra *T*_*e*_. In Fig. 2(g) *T*_*e*_ is shown as a function of the laser intensity *a*_0_. At *a*_0_ = 5 the electron temperatures obtained with the two different plasmas are comparable, whereas at *a*_0_ = 45 the homogeneous plasma *T*_*e*_ can exceed three times the foam one (72 MeV vs. 23 MeV, C-pol).

Direct laser acceleration of the electrons in a plasma channel is a resonant process^1,60^. The particles in the tail of the electron energy distribution are those for which the resonance condition is met, so that they stay longer in phase with an accelerating electromagnetic field. It is reasonable to expect that perturbing the electromagnetic field distribution would result in a detuning of the resonance condition. Figure 2(h and i) show the electromagnetic field inside the plasma channel for a0 = 45, for a uniform plasma and for a DLCCA-plasma respectively. In the case of the nanostructured plasma, the diameter of the channel is larger and the field is less intense and significantly more disordered.

We also consider the effect of the laser polarization. Figure 2(c,d,e,g) show that with homogeneous plasmas there is a non negligible polarization dependence of the energy absorption and the electron spectra. For instance, the highest electron temperature difference is ∼14% for *a*_0_ = 45 and the largest cut-off energy discrepancy is $\sim \mathrm{30 \% }$ for *a*_0_ = 15. On the other hand, all these differences are almost completely removed with nanostructured plasmas. In a simplified picture, this could be attributed to the fact that a laser pulse interacting with a nanostructured target is focused onto a non-flat surface. A similar effect was also observed in previous works^12,13^. We remark that the homogeneous plasma case for *a*_0_ = 5, C-pol was not considered in this discussion, since it is opaque to the laser pulse so that the interaction is qualitatively different from the other cases: with C-pol and normal incidence, electron heating mechanisms are suppressed, leading to a more efficient formation of a denser layer at the vacuum-plasma boundary with respect to P-pol.

### Effect of different nanostructure morphologies at low laser intensity

In order to assess the effect of different nanostructure morphologies on laser-plasma interaction, we performed three additional 3D simulations with laser intensity *a*_0_ = 5 and linear polarization, considering a DLA-foam, a DLCCA-foam, an ordered nanowires array and a random nanowires assembly as targets (see Fig. 1(a)).

**Figure 3:**
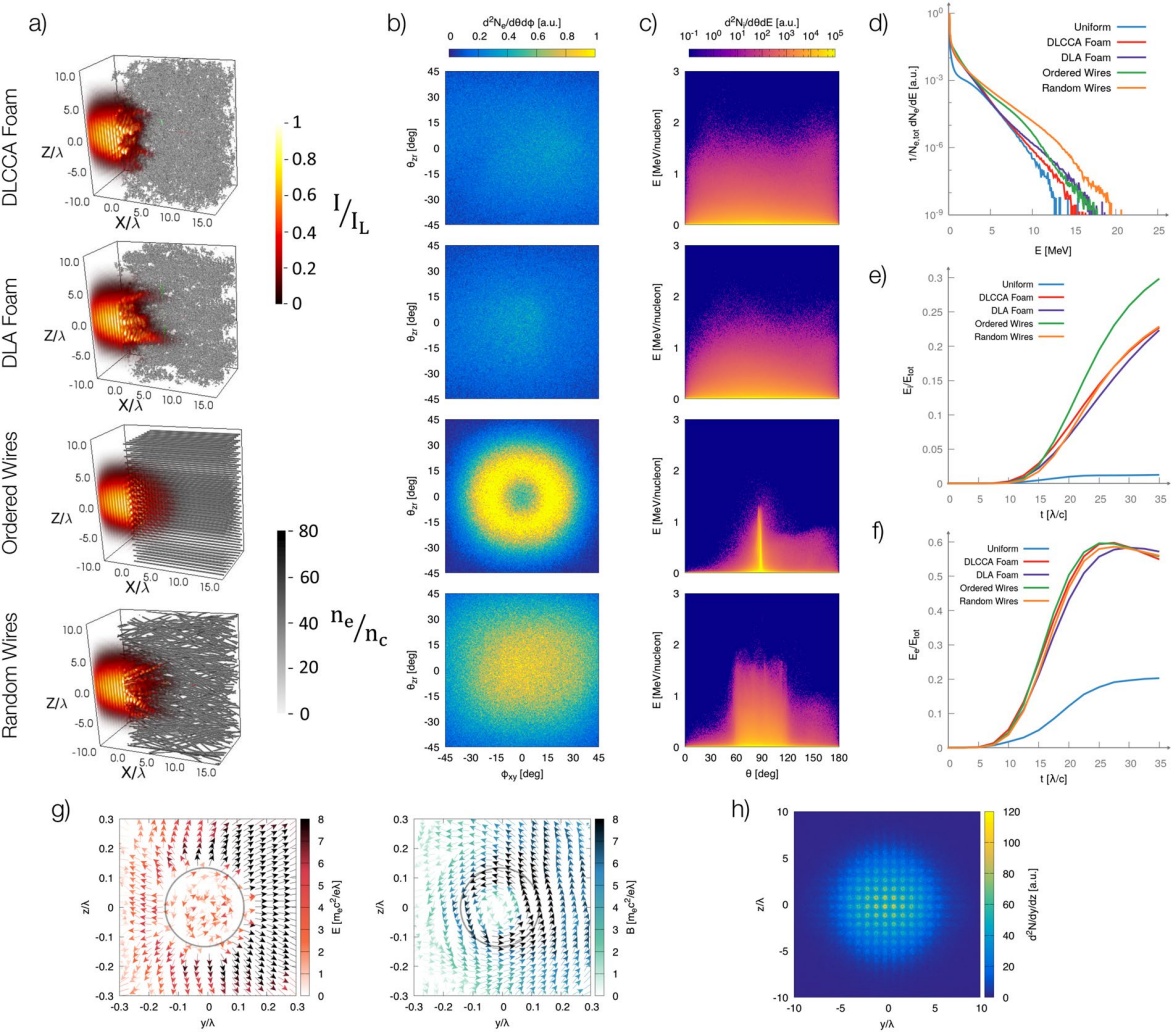
(**a**) Laser interacting with (from top to bottom): a DLCCA-foam, a DLA-foam, an ordered wires array and a random wires assembly (*t* = 15*λ*/*c*). The electron density is shown in grey scale, while the energy density of the electromagnetic field is represented in color. (**b**) Angular distribution of the propagation direction of energetic electrons (*E* ∈ [4, 4.5] MeV) at *t* = 30*λ*/*c*. *θ*_*zr*_ = 0°,*ϕ*_*xy*_ = 0° corresponds to forward propagation along $\hat{x}$ direction. (**c**) Distribution of ions as a function of the emission angle *θ* with respect to $\hat{x}$ and of the kinetic energy at time 30*λ*/*c*. *θ* = 90° corresponds to the transverse direction with respect to laser axis. (**d**) Electron energy spectra at *t* = 30*λ*/*c*. (**e,f**) Fraction of the laser energy converted respectively into ion and electron kinetic energy as a function of time. (**g**) Cross section (*x* = 15*λ*) of the electric and magnetic fields surrounding a wire of the ordered array at *t* = 30*λ*/*c*. The arrows indicate the direction of the fields on the plane, while the color is proportional to $\sqrt{{E}_{y}^{2}+{E}_{z}^{2}}$ (left plot, red color scale) or $\sqrt{{B}_{y}^{2}+{B}_{z}^{2}}$ (right plot, blue color scale). The grey circle marks the initial boundary of the wire. (**h**) Transverse projection of the density of energetic macro-particles for the ordered nanowire array. Electrons with kinetic energy 4 MeV < *E* < 5 MeV and position 10*λ* < *x* < 20*λ* are shown at *t* = 30*λ*/*c*.

Figure 3(a) shows the propagation of the laser pulse in all the simulated nanostructured targets. The first important observation is that the qualitative features of laser-plasma interaction do not significantly differ between each other. In particular, the penetration depth of the laser in the target is similar. For both nanowires targets a strong z-pinch^54^ is observed, due to the large currents induced along the wires. This effect is not observed for the two foam targets.

As reported in the previous section, a nanostructured target leads to a strong increase of the absorption efficiency with respect to a homogeneous plasma (see Fig. 3(e and f)). However, the effect of the nanostructure morphology on the total absorption efficiency was observed to be surprisingly mild. At *t* = 35*λ*/*c*, the absorption efficiency of laser energy into particle kinetic energy is $\sim \mathrm{75 \% }$ for all the disordered nanostructures (random wires, DLA-foam and DLCCA-foam), while being slightly higher ($\sim \mathrm{85 \% }$) for the ordered wires array. As shown in Fig. 3(e), this discrepancy reflects into a higher absorption efficiency of laser energy into ion kinetic energy for the ordered wires array ($\sim \mathrm{30 \% }$ instead of $\sim \mathrm{20 \% }$ as for the other nanostructured targets). More significant discrepancies are observed in the electron energy spectra, as shown in Fig. 3(d), with differences in the absolute number of high-energy electrons greater than one order of magnitude. The highest number of particles in the tail of the spectrum was observed for the random wires array. The cut-off of the electron energy distribution was found to depend only mildly on the nanostructure morphology, being always in the range 15–20 MeV.

Target morphology was observed to significantly affect the angular distribution of both energetic electrons and ions. As shown in Fig. 3(b), foam and random wires targets lead to rather uniform angular distributions of the emitted electrons. On the contrary, for the ordered wires array a ring-like structure appears, with a minimum on the laser axis and a maximum around $\sim 20^\circ$ (a factor of two in the intensity is observed between the minimum and the maximum of the distribution). This effect is likely due to the characteristic configuration of the electromagnetic fields for the ordered nanowire array (see Fig. 3(g)). The return current flowing inside the nanowires drives a strong azimuthal magnetic field, while charge separation at the wire surface induces a strong, radially outward, electric field. The effect of the magnetic field is to expel forward-moving electrons from the wire, while the electrostatic field is attractive (as shown in Fig. 3(h) energetic particles are most likely found in an annular region surrounding each wire). These fields hinder the propagation of electrons along the nanowires axis and the cylindrical symmetry of the distribution is a result of the cylindrical symmetry of the system. Figure 3(c) shows the distribution of the emitted ions. The foam-like targets lead to an emission with a weak angular dependence, while a strong peak at $\theta \sim 90^\circ$ (the transverse direction with respect to laser axis) was observed for the ordered nanowires. A uniform angular distribution between $\sim 90^\circ \pm 30^\circ$ was obtained with the random wires assembly (30° is the maximum tilt angle of the nanowires). This can be explained considering the cylindrical symmetry of plasma expansion for the nanowires target, which is radial for each one of them. A peak appears when they are all aligned along the laser axis, while the uniform angular distribution obtained with the random nanowires array reflects the uniform distribution of their tilt angle. In these conditions (relatively low laser intensities and relatively large nanoparticles) plasma expansion cannot be completely described as a Coulomb Explosion, showing also features better described with a fluid approach^61^.

### Effect of the dimensionality of the simulation

Numerical simulations are an important tool for the interpretation and the design of experimental activities. 3D simulations are usually necessary if a quantitative agreement between experimental and numerical results is desired. However, 3D simulations can be highly demanding in terms of computational resources. Understanding which features of laser-plasma interaction can be reliably reproduced in 2D could allow to perform numerical investigations with lower computational resources. This is especially beneficial if extensive parametric scans are required. The effect of the simulation dimensionality is particularly crucial for the case of a nanostructured target, since it might not be straightforward to reproduce in 2D an intrinsically 3D morphology. For this reason, for each 3D case, we performed a corresponding 2D simulation and we compared the main observables.

As far as a foam-like plasma is of concern, it is challenging to reproduce a 2D fractal-like structure with the same properties of its 3D analogous, keeping a similar filling factor and mean density. Thus, we used a random collection of nanospheres with the same radius, filling factor and density of those of the 3D case. Similar arguments apply for the nanowires targets, since in 2D it is not possible to reproduce at the same time the wire morphology (radius and spacing), the mean density and the local density. Thus we performed two simulations with different targets: one with the same spacing and wire radius of the 3D case but a higher density (∼12 *n*_*c*_), the other one with the same mean density of the 3D case (3 *n*_*c*_) but different geometry (thinner wires).

It should be highlighted that some physical processes such as charge separation, diffraction and self-focusing are inherently affected by the simulation dimensionality. For instance, in 2D geometry a charged particle is actually an infinite charged wire, thus the electrostatic potential is different from that of a charged particle in 3D. As a consequence, processes governed by charge separation, such as Coulomb explosion or Target Normal Sheath Acceleration, are expected to be heavily affected by the simulation dimensionality. We observed that the numerical results are indeed significantly affected by the simulation dimensionality. As a general feature, at fixed conditions, the pulse is able to propagate more deeply into the target in the 3D simulations. The laser absorption is rather similar (⪅10%) except for the *a*_0_ = 5 homogeneous plasma case. In this last case, in 3D the ponderomotive force of the laser creates a clean density spike at the pulse erosion front, which reflects the incoming beam. On the other hand, in 2D the erosion front exhibits modulations which probably boost laser absorption with respect to the former case ($\sim \mathrm{40 \% }$ in 2D, $\sim \mathrm{25 \% }$ in 3D). This effect is relevant only for plasmas close to the transparency threshold, since for highly transparent cases the laser penetrates deeply in the plasma and is efficiently absorbed regardless of the dimensionality.

We also report that the target morphology is crucial when studying nanowires in 2D. The plasma shaped to reproduce the 3D geometry resulted into an overdense material (∼12 *n*_*c*_), reflecting the laser beam within few wavelengths. On the other hand the 2D wires with a 3 *n*_*c*_ average density performed qualitatively similarly to the 3D case despite being much thinner.

Also the electron temperature is deeply influenced by the simulation dimensionality, being as far as twice the value in 2D with respect to the 3D case (the highest difference was found for the nanostructured plasma at *a*_0_ = 45).

In this work we also studied the laser self-focusing within a near-critical plasma. At this regard we define the self-focusing parameter $SF=({|{\bf{E}}|}^{2}+{|{\bf{B}}|}^{2})/{I}_{0}$ where **E** and **B** are the electromagnetic fields and *I*_0_ is the initial peak laser intensity. We always observed self-focusing except for *a*_0_ = 5, due to the low transparency of this case. We report a low dependence of SF with respect to the simulation parameters. In 3D simulations SF was within the 4.5–6 range, regardless of the laser polarization or the target structure. As also reported in previous works^1^, the SF is lower in 2D simulation, being ∼3, since focusing can only occur along a single transverse direction instead of in a whole plane.

## Discussion

In this work, we observed that the interaction of a relativistic laser pulse with a nanostructured NCP is significantly different than with a homogeneous one. Moreover, the morphology of the nanostructure was found to affect the interaction.

At moderate laser intensity (*a*_0_ = 5), a strong enhancement of absorption efficiency with respect to the homogeneous plasma case is reported, regardless of the morphology. This suggests that the use of low-density nanostructured materials could be beneficial for applications strongly relying on absorption efficiency (e.g. laser-driven ion acceleration). In the literature, aligned nanowire arrays have been shown to lead to very high absorption efficiencies. Here we show that similar performances can be obtained also with foams and random nanowire assemblies. This enhancement was found to be milder for *a*_0_ = 15 and negligible for *a*_0_ = 45. However, in all the investigated cases, the presence of a nanostructure leads to a strong increase of the absorption efficiency of laser energy into ion kinetic energy, due to the explosion of the nanostructures.

At high laser-intensity (*a*_0_ = 45), a marked detrimental effect of the nanostructure was observed in the tail of the electron energy distribution, with a ~50% reduction of the cut-off energy with respect to the homogeneous plasma. Negligible differences were obtained for the total absorption efficiency, however the target nanostructuring leads to an order of magnitude increase of the efficiency of energy transfer to the ion population. These results are relevant for experimental schemes aiming at the realization of high-energy synchrotron sources based on laser interaction with a NCP, since the energy of the emitted photons strongly depends on the energy of the electron population.

The morphology can strongly influence the angular distribution of energetic particles in laser interaction with nanostructured NCPs (this effect has been investigated only for *a*_0_ = 5). In particular, using an ordered array of aligned nanowires instead of random nanostructures leads to a ring-like shape of the angular distribution of emitted electrons and to an ion emission strongly peaked near the transverse direction with respect to laser axis. The latter effect could find application as a possible diagnostic of the integrity of a nanowire array during an actual experiment. Indeed the observation of a peak in the angular distribution of emitted ions at 90° from laser axis would be a strong signature of the survival of the nanostructure for long enough to interact with the main pulse.

As an important remark, we have observed that, although 2D simulations are able to qualitatively reproduce the 3D results, the simulation dimensionality may deeply affect the physical processes at play. As an example, at low laser intensity the electron temperature is comparable between 2D and 3D cases despite a much different laser absorption. Moreover, reproducing the nanostructure morphology at lower dimensionality was found to be by no means straightforward.

In conclusion, our results show that including a realistic description of the nanostructure is essential to properly model physical processes of direct relevance both for a satisfactory understating and for the foreseen applications of laser interaction with nanostructured NCPs. Moreover, they suggest possible paths to guide the design of future experimental activities.

## Methods

### Particle-In-Cell simulations

Particle-in-Cell^49^ simulations were performed with the open-source, massively parallel code *piccante*^62^.

The laser pulse had cos^2^ temporal profile and a Gaussian transverse profile and it was focused at the vacuum-plasma boundary. The waist was 5*λ*, while the temporal duration was 15*λ*/*c* (full-width-half-maximum of the fields). The intensity was varied between *a*_0_ = 5 and *a*_0_ = 45 at fixed normal incidence. These parameters, if scaled to Ti:Sapphire lasers, correspond to a 40 fs beam focused into a 4 *μ*m spot, providing a peak intensity in the range 5 ⋅ 10^19^ W/cm^2^ < I < 3.2 ⋅ 10^21^ W/cm^2^. Corresponding simulations with different polarizations are performed keeping the pulse energy constant. The reported value of *a*_*0*_ always refers to P-polarization and should be divided by 2^1/2^ for C-polariazion.

For the 3D simulations with a uniform plasma, a 90*λ* × 50*λ* × 50*λ* box was used, with a resolution of 20 points per wavelength. A uniform plasma slab filled a 50*λ* × 50 *λ* × 50*λ* region with a density of 3*n*_*c*_. 12 macro-electrons per cell were used.

For the 3D simulations with a nanostructured plasma, a 80*λ* × 30*λ* × 30*λ* box was used, with a resolution of 50 points per wavelength. The plasma filled a 40*λ* × 30*λ* × 30*λ* region (30*λ* × 30*λ* × 30*λ* region-thick for the wire targets) with an average density of 3*n*_*c*_. The local density was $\sim 60{n}_{c}$, with a filling factor of $\sim \mathrm{5 \% }$ (the local density was adjusted to have an average density of exactly 3*n*_*c*_). 64 macro-electrons per cell were used.

We performed additional convergence tests to ensure that both the spatial resolution and the number of macro-electrons per cell are adequate for our physical scenario. The case *a*_0_ = 45, P-polarization, homogeneous plasma was selected and simulations with resolution of 20, 30 and 40 points per wavelength were carried out. Negligible differences were observed for the electron energy spectra and for the absorption efficiency in these cases. Then, the case DLCCA-foam, P-polarization, *a*_0_ = 45 was simulated in a reduced box (40*λ* × 6*λ* × 6*λ*) with a plane wave laser, using either 64 or 128 macro-electrons per cell. No significant difference was found in the observables relevant for this work.

In the 2D simulations with a uniform plasma, a 160*λ* × 70*λ* box was adopted with a resolution of 100 points per wavelength. The uniform plasma (100*λ* × 70*λ* with a 3*n*_*c*_ density), was sampled with 4 macro-electrons per cells. The nanostructured plasma was obtained through a random ensemble of nanospheres with a radius of 0.05*λ* and density 60 *n*_*c*_. This corresponds to a filling factor of $\sim \mathrm{5 \% }$ and a volumetric mean density of 3*n*_*c*_. This plasma was sampled with 169 electrons per cell.

In all the simulated cases the plasma was fully pre-ionized and the the charge/mass ratio of the ions was 0.5 (e.g. *C*^6+^). The electron population was initialized with a small temperature (few eVs) to avoid numerical artifacts. In order to verify that this choice does not affect our results, we performed an additional simulation with a high initial temperature (∼1 keV) in a reduced box. We observed only a small blurring of the nanostructures and negligible differences for the observables of interest in our work.

### Nanostructured foam models

The DLCCA-foam structure is generated through an extension of the Diffusion-Limited Cluster-Cluster Aggregation model (DLCCA), built to simulate the structure of fractal aggregates, such as colloids and soot^63^. In its original version, the DLCCA model starts with a collection of monodisperse spherical particles, randomly disposed into a cubic mesh. Then, these particles can move in random directions, to simulate the Brownian diffusing motion; once the spheres touch each other, they stick together to form a rigid dimer, which can undergo random motion as well. This mechanism is repeated until all the particles in the box aggregate together in a large fractal structure. In this work, the DLCCA model is used to produce aggregates with different particles numbers, which follow an exponential distribution. Thus, the aggregates are vertically deposited onto a substrate to form the foam structure. The spheres are generated on a regular lattice with a radius of 0.0375*λ*, barely touching each other. In order to obtain a more realistic structure, the spheres are inflated up to a radius of 0.05*λ* and randomly displaced by a small fraction of their radius.

The DLA foam is obtained with a simple Diffusion-Limited Aggregation (DLA) model on lattice^59^ that works as follows. Particles are injected into a 3D uniform lattice from random sites located on the top of the lattice itself. Then, they are allowed to randomly walk in the lattice until they are halted because either they occupy a point adjacent to a pre-formed cluster or they ended up onto the bottom of the lattice, i.e. the substrate. Periodic conditions were used at the lateral boundaries. Using the same parameters (sphere radius and box size) as those used to generate the DLCCA foam we obtained DLA foams with much lower filling factors. For this reason, at first the DLA foam was generated with sphere particles with reduced radius (0.035*λ*) that then were inflated up to the desired radius (0.05*λ*). In this way we could obtain a filling factor value comparable to that of the DLCCA foam (~5%).

Both foams are generated in a 40*λ* × 6.4*λ* × 6.4*λ* box, which is then repeated periodically in the whole simulation box.

Nano-wire targets are modelled as a collection of cylinders, with a radius of 0.12*λ* (i.e. $\sim 100$ nm for a Ti:Sapphire laser, since in this case *λ* = 800 nm). Nanowires with similar properties and with a length of tens of *μ*m have been reported in the literature^39^. For the parallel wires target, the cylinders are aligned with the laser axis, while for the random wires target, their inclination angle is extracted randomly from a uniform distribution between −30° and +30° with respect to laser axis. The wires are truncated abruptly at the boundaries of the plasma region.

### SEM images of low-density foam aggregates

Carbon Foams showed in Fig. 1(c) are produced through the Pulsed Laser Deposition (PLD) technique using the parameters described by Zani *et al*.^41^. To obtain the images, the Zeiss Supra 40 field emission Scanning Electron Microscope (SEM) is exploited with an accelerating voltage of 5 kV.

### Data availability

The data supporting the findings of this work are available from the corresponding authors on request.
